# Advanced synchronous rectal and prostate cancers diagnosed by lateral lymph node dissection: A case report

**DOI:** 10.1016/j.ijscr.2021.106494

**Published:** 2021-10-12

**Authors:** Mizunori Yaegashi, Koki Otsuka, Yuya Nakamura, Tomoki Hatanaka, Kiyoharu Takashimizu, Akira Sasaki

**Affiliations:** a Department of Surgery, Iwate Medical University School of Medicine, Shiwa, Japan; b Department of Surgery, Iwate Prefectural Kuji Hospital, Kuji, Japan

**Keywords:** LLN, lateral lymph node, PSA, prostate-specific antigen, CT, Computed tomography, Lateral lymph node dissection, Prostate cancer, Rectal cancer, Synchronous cancer, case report

## Abstract

**Introduction:**

Rectal and prostate cancers are common cancers occurring globally, and both can metastasize to the pelvic lateral lymph nodes (LLNs).

**Presentation of case:**

A 69-year-old man, presenting with blood in stool, was diagnosed with rectal cancer. Computed tomography revealed a 7-mm LLN in the right internal iliac artery region, leading to the suspicion of metastasis. The patient underwent laparoscopic low anterior resection and LLN dissection. Histopathological findings of the metastatic tissue in the LLN were different than that of rectal cancer, and endocrine tumor was suspected. Immunostaining performed based on high serum prostate-specific antigen (PSA) level revealed positivity for PSA and α-methylacyl-CoA racemase in the dissected LLN. Thus, he was diagnosed with synchronous rectal and prostate cancers and received hormonal therapy for stage IV prostate cancer, which led to a dramatic reduction in PSA level after three months. He was followed regularly and did not relapse or experienced disease progression for either cancer for approximately four years after the initial diagnosis.

**Discussion:**

Few studies reported synchronous rectal and prostate cancers, both of which can metastasize to pelvic LLNs. However, preoperative diagnosis of the primary cancer metastasizing to the LLNs is challenging. Treatment of synchronous rectal and prostate cancers requires a strategy to diagnose each tumor stage and corresponding degree of progression because lymph node metastases affect staging in both cancers.

**Conclusion:**

Lymph node dissection may be useful in determining progression and treatment plan in cases of concurrent rectal and prostate cancers with suspected LLN metastasis.

## Highlights

• Lateral lymph node (LLN) metastasis is rarely reported in synchronous rectal and prostate cancer.
• LLN metastasis impacts staging in synchronous rectal and prostate cancer.
• Preoperative identification of the origin of LLN metastasis is difficult in such cases.
• LLN dissection may be useful to assess progression and treatment of synchronous cancers.

## 1 Introduction

Treatment of synchronous rectal and prostate cancers depends on the stage of both tumors [1]. Both tumors can spread to lateral lymph nodes (LLNs) in the pelvis [2], [3], [4], [5]. However, preoperative diagnosis of the primary tumor responsible for LLN metastasis is difficult in patients with synchronous rectal and prostate cancers. We herein present the case of a patient with synchronous rectal and prostate cancers, in whom LLN dissection was useful in diagnosing the primary tumor leading to the LLN metastasis. This case report was prepared according to the SCARE Criteria [6].

## 2 Presentation of case

**Fig. 1:**
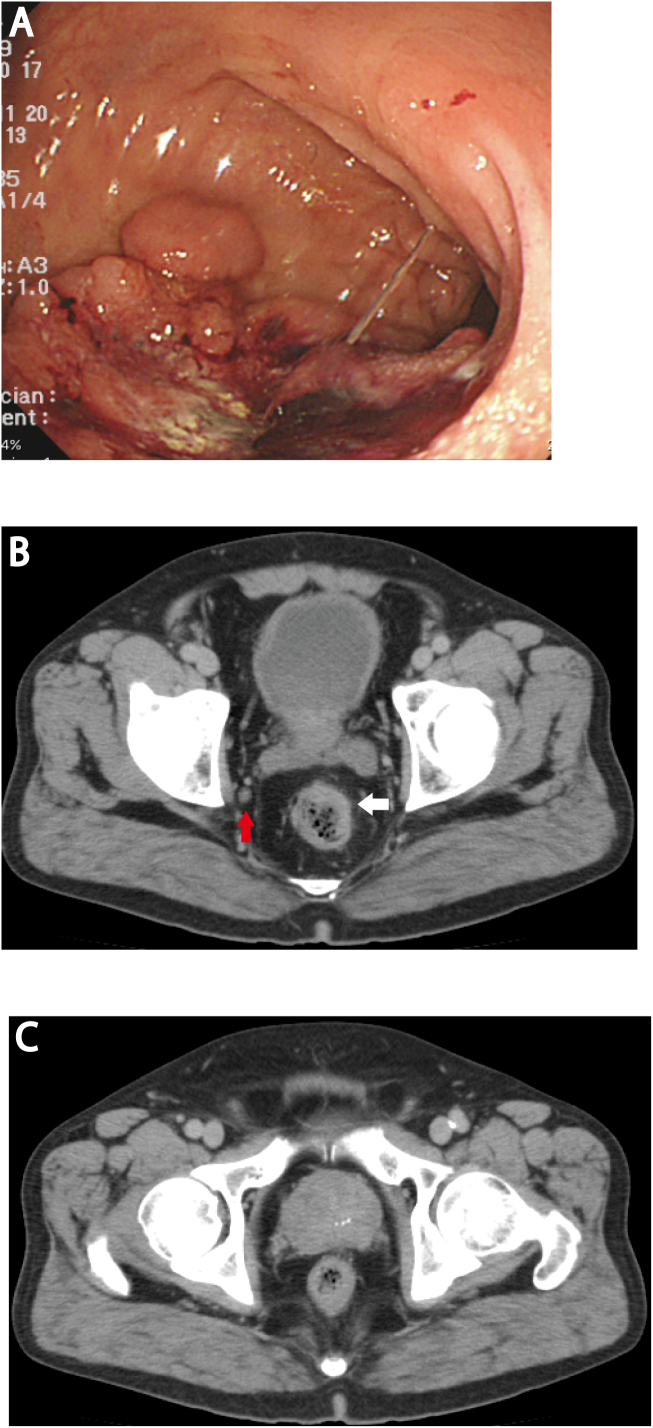
(A) Colonoscopy shows localized tumor in rectum at the time of diagnosis. Computer tomography shows (B) rectal cancer with increased rectal wall thickness (white arrow) and an enlarged lateral lymph node (diameter, 7 mm; red arrow) located in the right internal iliac artery area, and (C) calcification in the prostate gland.

A 69-year-old male patient presented to our institution complaining of blood in his stool. He had a history of hyperuricemia and cholecystolithiasis. He had no drug or allergy history, and had not had a recent medical checkup. A digital rectal examination showed no abnormalities, and he had no symptoms of prostate problems. A colonoscopy and a barium enema examination showed a rectal tumor (Fig. 1A). Histopathological examination of the biopsy specimen revealed a moderately differentiated adenocarcinoma. Other biochemical parameters, including serum carcinoembryonic antigen and carbohydrate antigen 19–9 levels, were within normal limits. Prostate-specific antigen (PSA) was not measured preoperatively. Computed tomography (CT) revealed a rectal tumor, increased rectal wall thickness, and an enlarged pelvic LLN in the right internal iliac artery area (Fig. 1B). There were no metastases in distant sites, including the liver, lungs, and bone. CT did not show any abnormalities in the prostate. Therefore, stage IIIC rectal cancer (T3, N2, M0) was diagnosed according to the TMN Classification of Malignant Tumors by the Union for International Cancer Control (8th Edition). The patient underwent laparoscopic low anterior resection, LLN dissection, and temporary ileostomy. The operation was performed by a gastrointestinal surgeon with >10 years of surgical specialty training. There were no postoperative complications, and he was discharged on postoperative day 17.

**Fig. 2:**
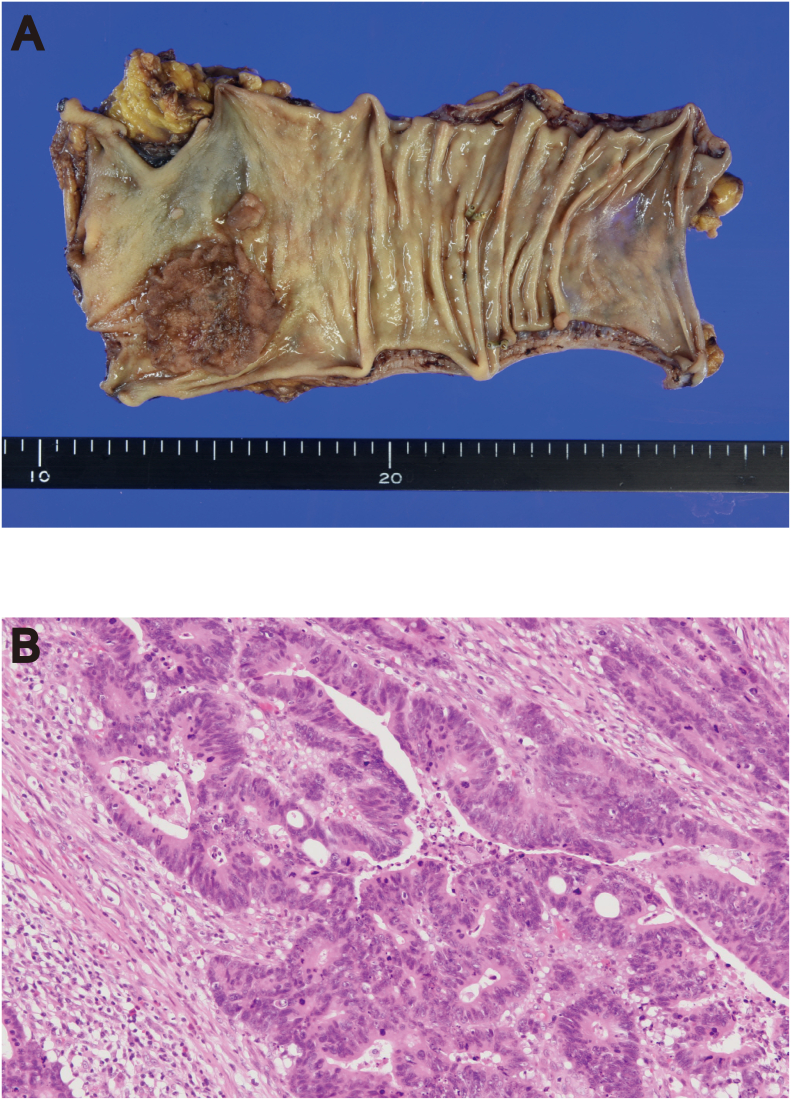
(A) Resected tumor in the rectum. (B) Histopathological examination of the rectal tumor showing moderately differentiated adenocarcinoma (hematoxylin/eosin staining; magnification, ×20).

**Fig. 3:**
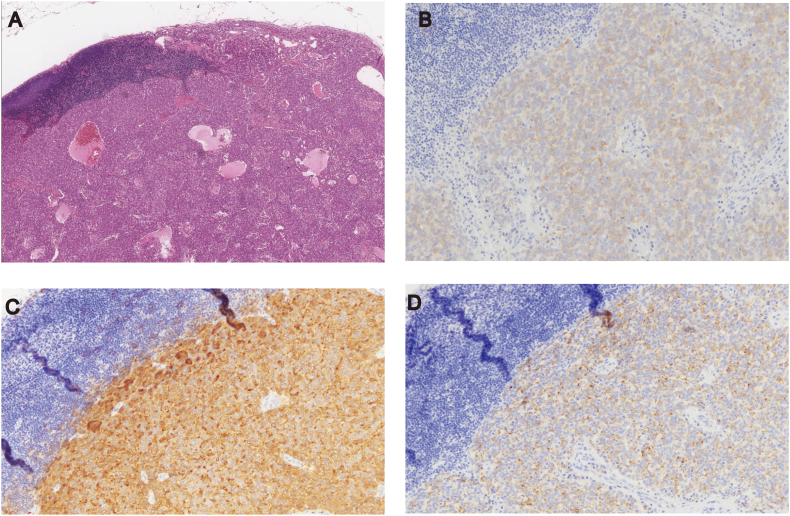
(A) Histopathological examination of the lateral lymph node in the right internal iliac artery area shows glandular structure with tumor cells exhibiting circular nuclei (hematoxylin/eosin staining; magnification, ×4). (B) Immunohistochemical staining shows positivity for synaptophysin (magnification, ×20), (C) prostate-specific antigen (magnification, ×20) and (D) α-methylacyl-CoA racemase (magnification, ×20).

The histopathologic diagnosis was rectal carcinoma based on the primary tumor specimen (Fig. 2). Lymph nodes within the mesorectum and mesentery of inferior mesenteric artery area did not show evidence of rectal cancer metastasis. The LLN specimen from the right internal iliac artery area showed malignant cells that were different from the primary rectal cancer (Fig. 3A). The malignant tissue was initially considered as an endocrine tumor based on the positive immunohistochemical staining for synaptophysin (Fig. 3B). However, the patient's postoperative serum PSA level was high (55.3 ng/mL). The dissected LLNs tested strongly positive PSA and α-methylacyl-CoA racemase on immunohistochemical analysis, confirming the presence of metastasis from the prostate (Fig. 3C, D). These findings led to the diagnosis of synchronous rectal and prostate cancers with final stages of IIA (T3, N0, M0) and IV (TX, N1, M0), respectively, according to the 8th edition of the TNM Classification of Malignant Tumors by the Union for International Cancer Control.

The patient received treatment with bicalutamide (80 mg once daily) and leuprorelin (22.5 mg once every 6 months) for prostate cancer, and his PSA levels decreased to 1.9 ng/mL three months later. For rectal cancer, the patient underwent ileostomy closure two months after the index surgery. He was followed regularly and did not relapse or experience disease progression for either cancer for approximately four years after the initial diagnosis.

## 3 Discussion

In 2018, colorectal and prostate cancers were, respectively, the third and fourth most common cancers worldwide, with 1,800,000 and 1,300,000 new cases annually [7]. Anatomically, pelvic LLN metastasis occurs in rectal and prostate cancers [2], [3], [4], [5]. Reportedly, coexistence of both cancers in the LLNs is rare [8]. However, identifying which primary cancers caused the lymph node metastasis in preoperatively is challenging in cases of synchronous rectal and prostate cancers. In Western countries, preoperative chemoradiation therapy has become a standard treatment option to reduce local recurrence rate. On the other hand, LLN dissection, which has also been shown to be an effective treatment approach for suppressing local recurrence after surgery for rectal cancer, is commonly performed in Japan [9]. In the present case, low anterior resection and LLN dissection were performed for rectal cancer with suspicious lymph node metastasis. The histopathological examination revealed that the metastatic cells in the dissected lymph node were distinct from rectal cancer cells, hindering definitive diagnosis. Prostate cancer was not initially considered based on the preoperative CT findings. However, the patient was subsequently diagnosed with prostate cancer because of high postoperative serum PSA level and positive immunostaining of the dissected LLN with PSA and α-methylacyl-CoA racemase, illustrating that LLN dissection was useful for the diagnosis of the primary tumor metastasizing to the LLN. Several reports have stated that prostate cancer screening reduced mortality [10], [11]. In Japan as well, screening using PSA is being conducted, and the number of deaths from prostate cancer has been gradually declining. On the basis of this experience, we suggest preoperative PSA measurement in male patients with suspected pelvic lymph node metastases and prostate disease [12]. In addition, prostate-specific membrane antigen-targeted positron-emission tomography may have contributed to the diagnosis of prostate cancer in this case [13].

Treatment of synchronous rectal and prostate cancers requires a strategy to diagnose each tumor stage and corresponding degree of progression, because lymph node metastases impact staging in both cancers. Therefore, it is critical to correctly identify the primary cancer metastasizing to the LLN in patients with synchronous rectal and prostate cancers. Prostate is richly supplied with lymphatics, which show LLN metastasis because of drainage to the LLNs [5]. Few studies reported LLN metastasis in synchronous rectal and prostate cancers. Therefore, as evidenced in our case report, lymph node dissection may be a useful intervention for diagnosing a primary tumor for lymph node metastases in the cases of synchronous rectal and prostate cancers with suspected LLN.

## 4 Conclusion

Prostate cancer should be suspected in patients with rectal cancer in the presence of LLN metastasis harboring findings that are distinct from those associated with rectal cancer. LLN dissection may be useful in determining disease progression and treatment for both cancers in synchronous rectum and prostate cancers with suspicious LLN metastasis.

## Ethical approval

Ethic approval has been exempted by Clinical Research Ethics Committee of Iwate Prefectural Kuji Hospital.

## Funding

This research did not receive any specific grant from funding agencies in the public, commercial, or not-for-profit sectors.

## CRediT authorship contribution statement

MY and KO conceived the case presentation and drafted the manuscript.

MY and KT participated in the design of the case presentation.

MY, YN, and TH provided management of the patient.

KO and AS read and approved the final manuscript.

## Guarantors

Koki Otsuka, Mizunori Yaegashi

## Registration of research studies

Not applicable.

## Consent

Written informed consent was obtained from the patient for publication of this case report and accompanying images. A copy of the written consent is available for review by the Editor-in-Chief of this journal upon request.

## Provenance and peer review

Not commissioned, externally peer-reviewed.

## Declaration of competing interest

None.
